# The Inaugural NIH‐Wide Strategic Plan for Autoimmune Disease Research (Fiscal Years 2026–2030)

**DOI:** 10.1002/art.43411

**Published:** 2025-12-12

**Authors:** Victoria K. Shanmugam, Carmen Ufret‐Vincenty, Xinrui Li, Janine A. Clayton

**Affiliations:** ^1^ Office of Autoimmune Disease Research, Office of Research on Women's Health, Office of the Director, NIH Bethesda Maryland

## Introduction

Autoimmune diseases represent a growing public health challenge. Historical estimates of prevalence indicate that approximately 7% to 8% of the US population are affected.^1^ However, more recent estimates, including the more than 140 recognized autoimmune diseases, suggest that up to 50 million Americans are living with autoimmune disease.^2^, ^3^

These chronic, often debilitating diseases commonly present with systemic involvement, unpredictable flares, and complex comorbidities.^4^, ^5^ Despite decades of significant advances in immunology, genetics, and therapeutics, the burden of autoimmune disease remains high, with substantial unmet needs in diagnosis, management, and prevention.

Recognizing these challenges, the NIH has released its inaugural NIH‐Wide Strategic Plan for Autoimmune Disease Research, Fiscal Years 2026–2030. This plan marks an unprecedented cross‐institute approach for advancing autoimmune disease research. Here we provide an overview of the plan's development, priorities, and implications for the rheumatology and broader autoimmune disease communities.

## Background and rationale

Autoimmune diseases are a significant cause of chronic morbidity and mortality,^1^, ^6^, ^7^, ^8^ often heightening the risk of other chronic conditions such as cardiovascular disease.^4^ Although many autoimmune diseases are more common in women, men who develop these conditions often experience more severe symptoms and, in some cases, have a higher risk of mortality. Recent evidence indicates that the incidence and prevalence of these diseases are also increasing worldwide.^9^ In the US alone, the direct health care costs associated with autoimmune diseases surpass $100 billion annually. Additionally, indirect costs stemming from lost productivity and caregiver burden further elevate these estimates.^1^, ^10^, ^11^

With NIH support, substantial progress has been made in autoimmunity over the past 75 years. However, major gaps persist in understanding disease mechanisms, risk stratification, early detection, and development of curative therapies. Historically, autoimmune disease research has been significantly underfunded relative to the high and increasing prevalence of these conditions in the population, with most research and funding directed toward organ‐ and disease‐specific manifestations rather than addressing the broader phenomenon of autoimmunity. Although NIH support has allowed insights into shared pathogenesis in autoimmunity, there remain many unmet opportunities for developing crosscutting interventions and addressing the fundamental drivers of autoimmunity.

Autoimmune disease prevalence is rising faster than can be explained by genetics alone, and autoimmune diseases frequently co‐occur within individuals and families. These observations highlight the importance of studying common exposures across autoimmune diseases.^12^, ^13^ The 2022 National Academies of Sciences, Engineering, and Medicine report *Enhancing NIH Research on Autoimmune Disease* stressed the importance of a unified, multidisciplinary research agenda to accelerate scientific discovery in this area.^2^

## Plan development and engagement

Congressional directives in the Consolidated Appropriations Act, 2023, requested the establishment of the Office of Autoimmune Disease Research (OADR) in the NIH Office of Research on Women's Health and tasked the OADR with coordinating development of an NIH‐wide strategic plan, incorporating input from across the NIH, patient communities, clinicians, researchers, and advocacy organizations.

The plan's development was informed by the following sources:

- **Landscape analysis** of the NIH autoimmune disease research portfolio.
- **Request for information** and community roundtables to gather perspectives from patients, clinicians, and scientists.
- **Insights** from the 2022 National Academies of Sciences, Engineering, and Medicine consensus study.
- **Input from NIH institutes and centers (ICs)** and the Coordinating Committee for Autoimmune Disease Research, representing the NIH ICs.

The resulting plan integrates fundamental, translational, epidemiologic, clinical, and implementation science, aiming to catalyze innovation and collaboration across the autoimmune disease research continuum.

## Strategic priorities and crosscutting themes

**Figure 1:**
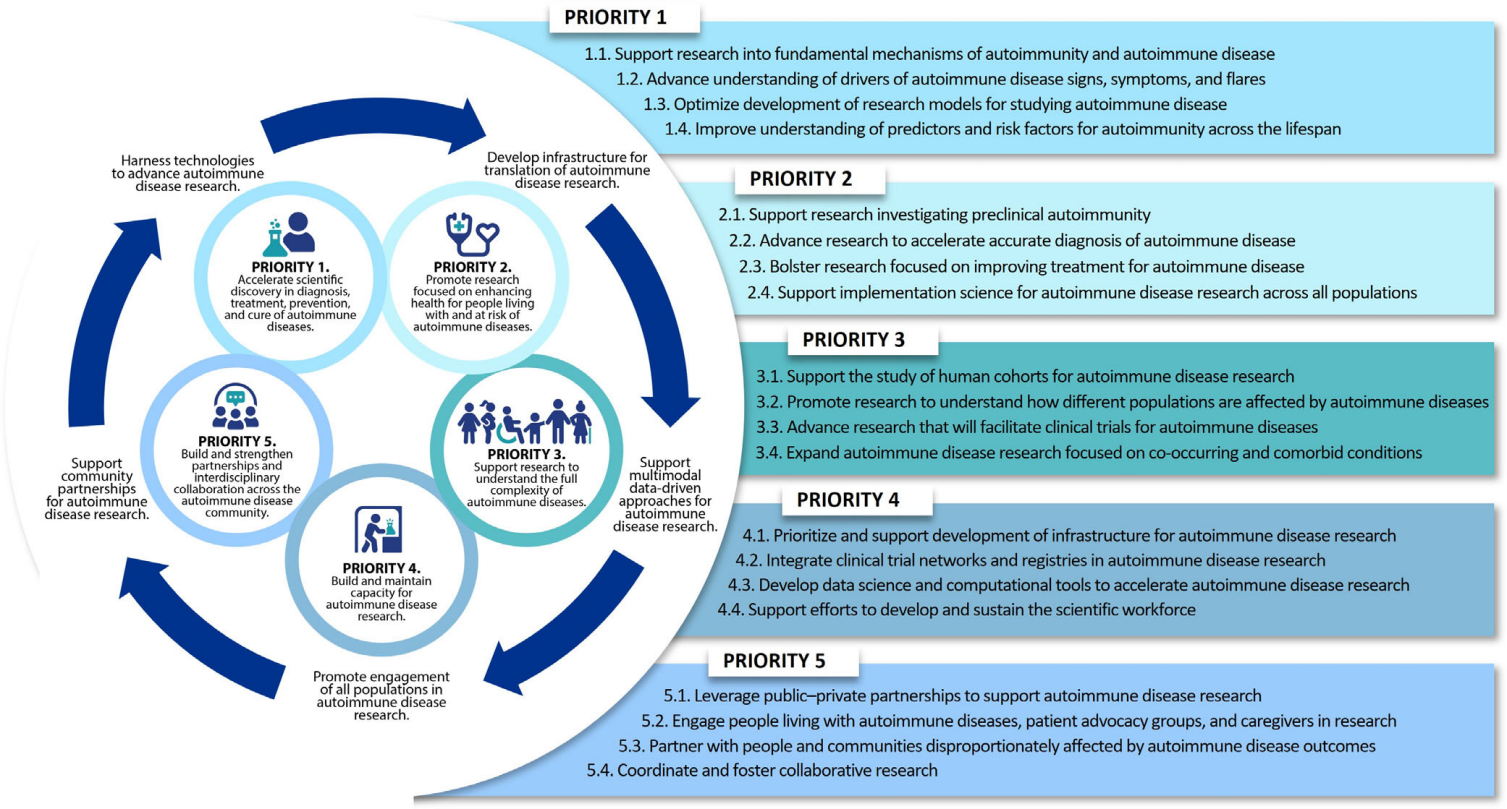
Schematic overview of the NIH‐Wide Strategic Plan for Autoimmune Disease Research (Fiscal Years 2026–2030): five strategic priorities and five crosscutting themes supporting a unified, collaborative research ecosystem spanning >140 autoimmune diseases and all NIH institutes and centers. The full NIH‐Wide Strategic Plan for Autoimmune Disease Research may be downloaded at: https://orwh.od.nih.gov/OADR‐ORWH/Strategic‐Planning‐for‐ADR.

The NIH‐wide plan is structured around **five strategic priorities**, each supported by specific objectives and **five crosscutting themes** (Figure 1).

1. **Accelerate scientific discovery in diagnosis, treatment, prevention, and cure of autoimmune diseases**. Advancing basic and translational research into the molecular and cellular mechanisms of autoimmunity is critical. Objectives under this priority include the following:
  - Supporting research into fundamental mechanisms of autoimmunity
  - Advancing understanding of drivers of autoimmune disease signs, symptoms, and flares
  - Optimizing development of research models for studying autoimmune disease
  - Improving understanding of predictors and risk factors for autoimmunity
2. **Promote research focused on enhancing health for people living with and at risk of autoimmune diseases**. Timely diagnosis and effective treatment remain key challenges for people living with and at risk for autoimmune diseases. Objectives under this priority include the following:
  - Research into preclinical autoimmunity
  - Advancing research to accelerate accurate diagnosis of autoimmune disease
  - Bolstering research focused on improving autoimmune disease treatments
  - Supporting implementation science for autoimmune disease research
3. **Support research to understand the full complexity of autoimmune diseases**. Understanding heterogeneity in disease manifestation, progression, and comorbidities across populations and across the lifespan is essential. Under this priority the plan calls for the following:
  - Supporting the study of human cohorts for autoimmune disease research
  - Promoting research to understand how different populations are affected by autoimmune diseases
  - Advancing research that will facilitate clinical trials for autoimmune diseases
  - Expanding autoimmune disease research focused on co‐occurring and comorbid conditions
4. **Build and maintain capacity for autoimmune disease research**. Investing in infrastructure and development of the scientific workforce are vital to sustain progress in autoimmune disease research. The objectives included in this priority are to do the following:
  - Prioritize and support development of infrastructure for autoimmune disease research
  - Integrate clinical trial networks and registries into autoimmune disease research
  - Develop data science and computational tools to accelerate autoimmune disease research
  - Support efforts to develop and sustain the scientific workforce
5. **Build and strengthen partnerships and interdisciplinary collaboration.** Developing and maintaining partnerships across the autoimmune disease community is crucial to stimulate innovation in research. The objectives included in this priority are to:
  - Leverage public–private partnerships, including those within industry, philanthropy, and other federal agencies
  - Engage patients, advocacy groups, and caregivers as partners in research
  - Partner with people and communities disproportionately affected by autoimmune disease outcomes
  - Coordinate and foster collaborative autoimmune disease research

## Crosscutting themes

Five crosscutting themes representing foundational concepts interwoven through the strategic plan were incorporated to complement the strategic priorities and objectives. These themes include:

1. Harness technologies to advance autoimmune disease research
2. Develop infrastructure for translation of autoimmune disease research
3. Support multimodal, data‐driven approaches for autoimmune disease research
4. Promote engagement of all populations in autoimmune disease research
5. Support community partnerships for autoimmune disease research

## Implementation vision

To implement the NIH‐Wide Strategic Plan for Autoimmune Disease Research, the NIH expects to use a range of funding mechanisms—such as investigator‐initiated awards (including those responsive to NIH highlighted topics available at https://grants.nih.gov/funding/find‐a‐fit‐for‐your‐research/highlighted‐topics), research funding opportunity announcements (which may be searched at https://grants.nih.gov/funding/nih-guide-for-grants-and-contracts), federal prize competitions, public–private partnerships, and cooperative agreements to maximize available resources and support various research approaches to ensure sustained progress. Some of the bold initiatives outlined in the plan include developing a scalable federated data infrastructure, investing in longitudinal immunophenotyping repositories, and supporting the research pipeline and biomedical workforce. In partnership with the scientific community, implementation of this strategic plan will build upon existing programs to accelerate autoimmune disease research innovation and scientific discovery.

## Implications and opportunities for autoimmune disease

The NIH‐Wide Strategic Plan for Autoimmune Disease Research marks a pivotal shift for the autoimmune disease research community, providing a unified, interdisciplinary research agenda for the next five years. By breaking down silos and encouraging cross‐disease insights, the plan will be particularly impactful for conditions with multisystem involvement, allowing identification of shared immunopathology and more comprehensive research approaches. Its focus on preclinical autoimmunity and prevention, especially through studying the prodrome, opens new avenues for early intervention. Improved understanding of risk factors, biomarkers, and environmental and molecular triggers, as well as attention to sex, age, and ancestry differences, will advance personalized care and monitoring.

The plan's commitment to data science promises to accelerate discoveries, reduce duplication, and enable more inclusive research. Emphasizing innovative clinical trial designs and developing clinical trial networks will also be essential to accelerating new therapies for autoimmune diseases. The plan also acknowledges looming workforce shortages in rheumatology and allied specialties and calls for initiatives to bolster training and career development across both the scientific and clinical workforce.^14^, ^15^

The inaugural NIH‐Wide Strategic Plan for Autoimmune Disease Research represents a transformative vision for the field. By integrating fundamental discovery with translational, clinical, and implementation science, as well as centering patient and community voices, the plan lays the foundation for accelerating progress toward improved diagnosis, management, and ultimately prevention and cure of autoimmune diseases. Continued, sustained funding will be required to match the growing burden of autoimmune diseases and realize ambitious goals articulated in this strategic plan. However, the plan offers a roadmap for how the NIH hopes to partner on collaborative, impactful work across diseases and disciplines to advance discovery for people living with and at risk of autoimmune disease.

## AUTHOR CONTRIBUTIONS

All authors contributed to at least one of the following manuscript preparation roles: conceptualization AND/OR methodology, software, investigation, formal analysis, data curation, visualization, and validation AND drafting or reviewing/editing the final draft. As corresponding author, Dr Shanmugam confirms that all authors have provided the final approval of the version to be published and takes responsibility for the affirmations regarding article submission (eg, not under consideration by another journal), the integrity of the data presented, and the statements regarding compliance with institutional review board/Declaration of Helsinki requirements.

## Supporting information


**Disclosure form**.
